# Pig tail length is associated with the prevalence of tail malformations but not with inflammation of the tail

**DOI:** 10.1186/s12917-025-04598-y

**Published:** 2025-02-26

**Authors:** Christiane Egerer, Katharina Gerhards, Sabrina Becker, Petra Engel, Sven König, Gerald Reiner

**Affiliations:** 1 Department of Veterinary Clinical Sciences, Clinic for Swine, Justus-Liebig-University, Frankfurter Strasse 112, Giessen, 35392 Germany; 2 Institute for Animal Breeding and Genetics, Justus-Liebig-University, Ludwigstrasse 21, Giessen, 35390 Germany

**Keywords:** Tail biting, Inflammation, Necrosis, Tail length, Malformations

## Abstract

**Background:**

Assuming that tail length is associated with the prevalence of tail biting, attempts are being made to shorten tails by genetic selection in order to avoid the painful procedure of docking. However, undesirable side effects such as kinky tails and inflammatory changes may occur. The aim of the present study was to clinically quantify in a population with known segregation of tail length, i) its variability, ii) possible associations with kinked tails and iii) possible associations of tail length and kinks with inflammation of the tail using 348 piglets at day 3 (undocked) and 39 (docked tails) of life.

**Results:**

The relative tail length (tail tip to tail base/tail tip to ear base × 100) varied between 20.3 and 31.3%. A reduced tail length was associated with kinked tails. Piglets with the shortest tails had 28% kinked tails, 5.6 times more than the piglets with the longest tails. The tails showed high prevalence of inflammation both on day 3 and on the docked tails on day 39. Overall, these were not associated with tail length or kinked tails. Only necrosis of the tail was significantly more frequent in the kinked tails than in the normal tails. Sow line, sow ID and boar ID significantly affected relative tail length, which may suggest a genetic cause.

**Conclusion:**

Based on the phenotypic variation found in the present study, it seems possible to influence tail length through breeding. It remains to be seen whether the available potential is sufficient to actually reduce tail biting. At the same time, a higher incidence of kinked tails and necrosis is to be expected.

**Supplementary Information:**

The online version contains supplementary material available at 10.1186/s12917-025-04598-y.

## Background

Tail docking has been used for decades as a means of combating tail biting [1, 2]. Although, tail docking can reduce biting considerably, it does not completely eliminate the problem, leaves the underlying causes unresolved and further increases damage, inflammation, pain, and animal welfare concerns [3, 4]. The procedure is painful and causes tissue damage, enhanced attempts to escape, high frequency vocalizations, pain associated behaviour, and elevated serum cortisol levels [5]. Additionally, the formation of traumatic neuromas through injured peripheral nerves at the tail tip might lead to chronic discomfort and dysesthesia [6], although there is some debate in the literature about this [7].

At the same time, discontinuation of tail docking can seriously increase the prevalence of tail lesions [8, 9]. Thus, there is still insufficient implementation of the existing tail docking ban in the EU (EU Directive 2008/120/EC) in most countries [2].

The risk of tail biting decreases with decreasing length of the docked tail [2, 10–12]. Two main causes are discussed as being responsible for this finding. On the one hand it is discussed that an increase in sensitivity in the remaining stump, which could be attributed to the formation of neuromas, might cause pigs to withdraw their tails more effectively and more quickly when biting attempts are made and thus to escape injury [6, 13–15]. Another effect discussed is that the shorter tail is less accessible and inviting to biting than the longer one [10, 14, 15]. As already discussed by Taylor et al. [12] and Simonsen et al. [14], the association between tail docking and the lower risk of tail biting still is not known, and there is still a considerable need for research to reduce tail biting in practice (for review see Henry et al. [16]). Evidence from research is there, but what lacks is practical knowledge on how it can be implemented in practice in a wide variety of farm conditions. Additionally, docked tails and shorter tails, do not reduce the risk of ear biting or flank biting, which are similar to tail biting [17].

The prospect of a correlation between tail length and biting has led to the approach of supporting the effort against tail biting by selecting pigs with shorter tails [18–20]. However, shorter tails can be accompanied by the appearance of malformations like kinked tails in various species [21–26] including pigs [18]. Other studies show that vasculitis, intimal proliferation and thrombus formation in the tail area can lead to reduced blood supply and promote the development of inflammation and necrosis as a further cause for tail damage besides tail biting [27–29]. It is conceivable that pathomorphological alterations associated with the appearance of shorter or even kinked tails could have additional effects on the integrity of the blood vessels and thus promote the occurrence of tail lesions.

Thus, the objective of the present study was to characterise variation in tail length in pigs in a population under selection for segregating tail length [20], i.e. with a higher variation in tail length than normally expected and to clinically study the possible associations between tail length with malformation (kinked tail) or inflammation and necrosis.

## Results

### Tail length

The piglets weighed between 0.5 and 2.7 kg (1.66 ± 0.40, Mean ± SD). The correlation between weight and absolute tail length was 0.712 (*R*^2^ = 50.8%; *P* < 0.001). Based on a regression constant of 6.35, absolute tail length increased by 1.90 cm per kg of body weight. Using a tape measure and following the whole tail from the tip to its base on the body exactly in its median, tails were 9.91 ± 2.11 cm long with a minimum of 6.26 cm and a maximum of 12.0 cm.

The relative tail length of the piglets was normally distributed with a mean of 25.5% and a standard error of 0.62. It varied between 20.3 and 31.3% (Min, Max). The correlation between body size and relative tail length was −0.05 (n.s.). Due to the strong dependence of the absolute tail length on the body size of the animals, all results presented in the following refer exclusively to the relative tail length (in %).

**Fig. 1:**
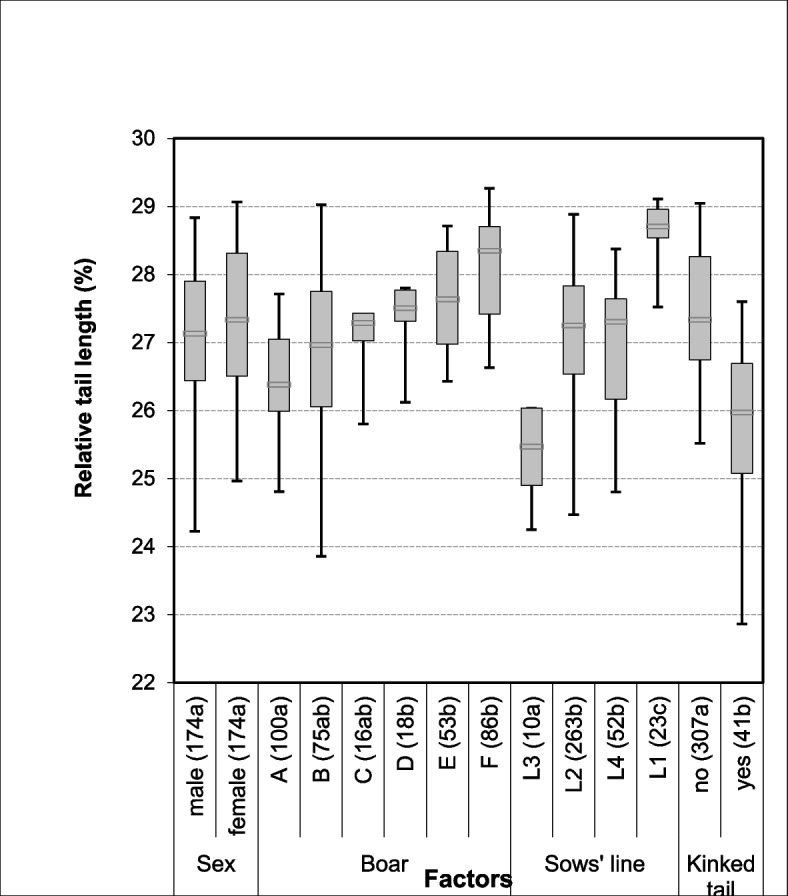
Relative tail length in percent (length from tail tip to tail base/length from ear base to tail tip*100) of 348 3-day old piglets by sex, boar, sow line and kinked tails. Median with boxes (percentiles 25 and 75) and whiskers (percentiles 5 and 95). The number of piglets is given in brackets in the x-axis legend, together with the superscript for the significance (within the same factor, characteristics with exclusively different letters are statistically significant (*P* < 0.05)

**Fig. 2:**
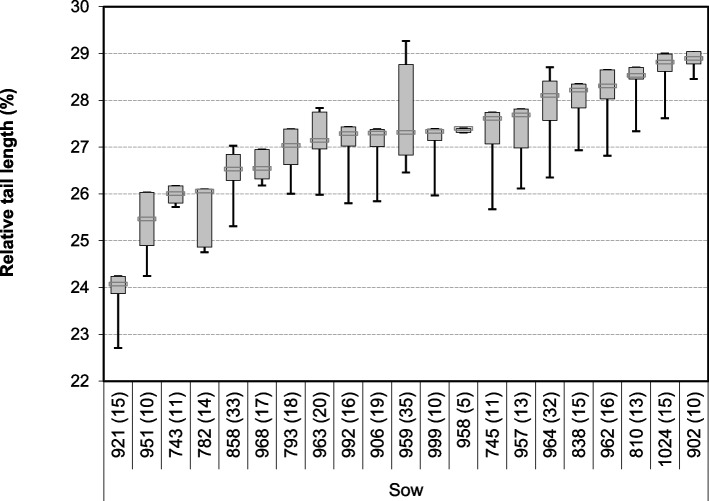
Relative tail length in percent (length from tail tip to tail base/length from ear base to tail tip*100) in 348 3-day old suckling piglets from 21 different sows (general linear model). The number of piglets is given in brackets in the x-axis legend. Significances between sows are given in Supplemental Table 1

The relative tail length was significantly affected by kinks (*P* < 0.001), boar (*P* = 0.004), sow line (*P* < 0.001) (Fig. 1) and sow (*P* < 0.001) (Fig. 2). Tails without kinks had a median relative tail length of 27.3%, those with kinks reached 25.9% of the body length. Piglets of different boars reached median relative tail lengths between 26.4 and 28.3%. Differences between some of the boars’ offspring were statistically significant. Piglets from sows of line 3 had median relative lengths of 25.5, those from sows of line 1 of 28.7%. The effect of birth weight and litter size were significant at *P* = 0.01 and *P* = 0.026, respectively. There was no effect of the sex and none of the parity. Means for relative tail length in the offspring of the 21 sows varied between 24.1 and 28.9% (Fig. 2).

### Kinked tails

**Fig. 3:**
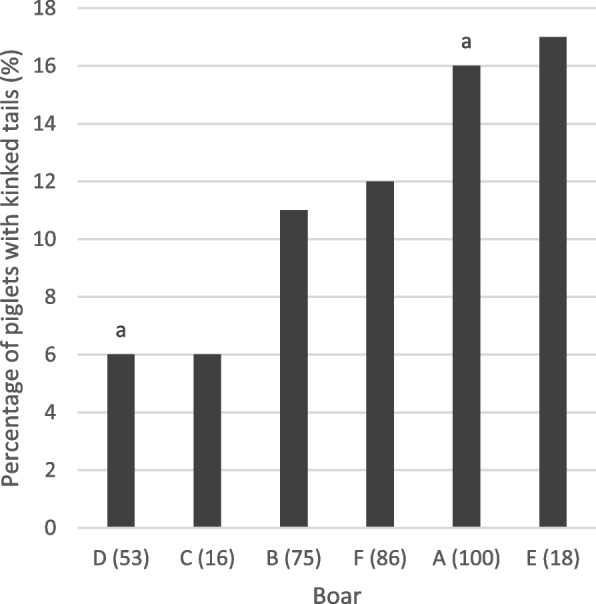
Percentage of piglets with kinked tails (%) by boar (A-F). Boars A and D are statistically significant (*P* < 0.05; generalised binary model. The number of piglets is given in brackets in the x-axis legend; total number: 348 piglets

**Fig. 4:**
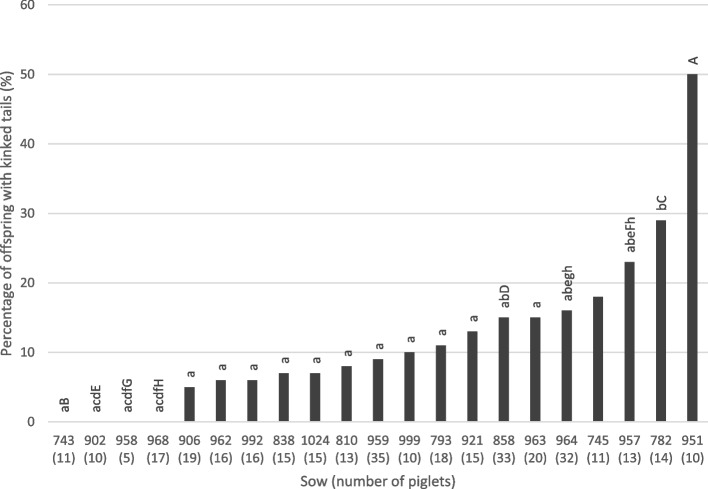
Percentage of piglets with kinked tails by sow (descriptive data from 348 piglets). Numbers of piglets per sow in brackets. Sows with small letters are significantly different from sows with the respective capital letter (*P* < 0.05)

Just under 12% of the tails examined were found to be kinked. Kinks of 30°, 60°, 90°, and 180° were found in 9.1%, 0.3%, 0.8%, and 0.6% of the tails. Offspring of boars C and D had the lowest prevalence with 6%, offspring of boars A and E the highest with 16% and 17%, respectively (Fig. 3). No kinky tails occurred in 4 of the 21 litters. On the other side, 50% of the litter was affected in sow number 951 (Fig. 4).

**Fig. 5:**
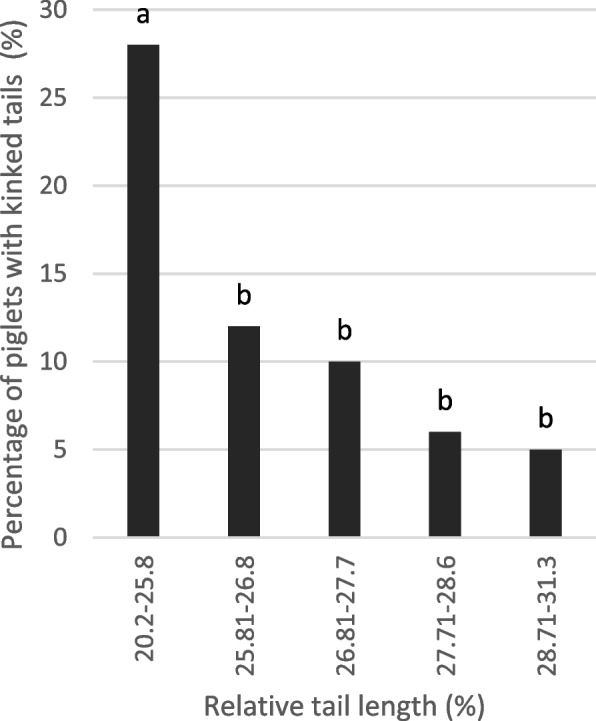
Percentage of piglets with kinked tails (%) by relative tail length (monofactorial linear model estimated with probit function). Each group contains 20% of all piglets (*n* = 66–67). The relative tail lengths (length from tail tip to tail base/length from ear base to tail tip*100) are shown as the labelling of the x-axis. Differences in percentage of kinky tails were significant (*P* < 0.001). Significant groups are marked with different letters

After equal division of all piglets into five groups of increasing tail length, a statistically significant (*P* < 0.001), negative correlation was found between tail length and the proportion of kinked tails in a monofactorial generalised linear model with probit function. Within the 20% piglets with the shortest tails (group 1) 28% had kinked tails (Fig. 5). In contrast, among the 20% piglets with the longest tails, only 5% of tails were kinked. The correlation between relative tail length and the incidence of kinked tails was −0.261 (*p* < 0.001).

Only five litters out of the 24 boar x sow-combinations were free from kinked tails. The relative tail lengths in litters with at least one piglet with kinked tail (*n* = 285) and litters without kinked tail (*n* = 63) were 27.2 ± 1.88% and 27.03 ± 1.61%, respectively. Thus, the relative tail lengths were the same in both groups. Within the litters with at least one piglet with kinked tail, the relative tail length was 27.4 ± 1.7% in piglets without kink (*n* = 244) and 25.9 ± 2.1% in piglets with kink (*n* = 41). This difference was statistically significant (*P* = 5.6 × 10^–7^).

### Inflammation and necrosis

Cases of tail biting were undetected, both in suckling piglets and weaners, neither by daily control, nor by the evidence of biting marks.

On day 3, the bristles at the tail base had fallen out in 71% of the piglets, 47% showed swelling, 22% redness, 2% exudation and 1% haemorrhages. On day 39, bristle loss had decreased to 46%, 36% showed redness, 21% swelling and 2% exudation each. On day 3, tail tip necrosis was already present in 9% of the piglets. On the docked tail tip, 15% of the piglets showed necrosis, 6% haemorrhages and 37% exudation on day 39. Between 46 and 57% of the piglets had bristle loss, redness or swelling. Despite considerable differences in the prevalence of inflammatory symptoms and necrosis between tail base and tail tip, no significant differences were found that could be attributed to tail length.

There were also no significant differences in the inflammation parameters between piglets with kinked tails and without. One exception was the occurrence of necrosis on both tail base and tail stump at day 39, which was significantly higher in kinked tails (29%) than in tails without this deformation (14%) (*p* < 0.05).

## Discussion

Tail docking is still widely used in many countries as a measure to reduce tail biting in pigs [2, 11], although it does not safely prevent biting and per se causes pain, suffering and damage to the affected piglets [3, 4]. It is not yet fully understood whether and to what extent the effect of docking is due to increased painfulness of the stump [6, 7, 13–15] and associated increased avoidance behaviour towards interacting pen mates or the reduced conspicuousness and attractiveness of the shortened tail [10, 14, 15]. Nevertheless, there are approaches to shorten the tail by genetic selection to achieve the presumed second aspect of docking, reduced attraction, without having to accept the pain and suffering associated with docking [18–20]. Apart from the fact that even this cannot solve the basic problem of active tail biting, which is made up of a highly complex sequence of very different feeding, husbandry and management conditions [5, 11–13], there are indications that selection for shortened tails could bring deformities in the form of kinky tails [18]. Furthermore, the integrity of the tail is threatened not only by biting but also by endogenous inflammation and necrosis (SINS, Swine Inflammation and Necrosis Syndrome [27]), which occur in close association with histopathologically detectable damage in the area of the upstream blood vessels [28–30]. Although it is not yet known whether pathomorphological alterations in the blood vessels can also occur in shortened or kinked tails, such a connection is quite conceivable.

This led to the approach of the present study, which had three objectives: i) to descriptively assess the variability of tail length in a population for which the segregation of this trait has already been described, ii) possible associations with kinked tails, and iii) possible associations of tail length and kinks with inflammation and necrosis at the base and tip of the tail. The evaluation was carried out by means of a multivariate model, taking into account the effects of boar, sow, sow’s line, sex and weight of the piglet as well as the number of piglets in the litter. In contrast to the study by Kunze et al. [18], neither the weight of the piglets, nor the litter size or the parity showed a significant effect on tail length. This difference results from the fact that the absolute tail lengths used in [18] are highly dependent on the size of the piglets ([18] and present study: *r* = 0.6), which are also influenced by litter size and parity, while the present study always uses the relative tail length (length from tail tip to tail base on body divided by body length from upper ear base to tail tip in percent) which is independent from piglets’ size. However, there were significant differences in tail lengths depending on the boar and sow (in concordance with [18]). This confirms the genetic component of tail length assumed for this population, which was calculated by [20] with a heritability of 0.42. But genetic component was not subject of the present study. The average values of the relative tail lengths of the piglets varied between 20.2 and 31.3%. Standardised to the average total length of the piglets on day 3 (ear base to tail tip) of 35 cm, this corresponds to absolute tail lengths of 7 to 11 cm. Further studies would have to show whether this difference of 36% between maximum and minimum tail length would have any influence on tail biting. Thodberg et al. [10] showed a largely linear relationship between tail length after docking and the prevalence of tail biting. Thus, a reduction in biting could already be achieved with a reduction in tail length from 7.5 cm undocked/docked to 5.7 cm docked (= −24%). However, due to the number of animals used, this difference only became statistically significant after the tail was shortened to 2.9 cm (= −61%).

The present study showed a close correlation between tail length and the occurrence of kink tails. With the largest tail lengths (group 5: > 28.7%), 5% kinky tails were recorded. From group 4 to group 2 (28.6 to 25.82% relative tail length) the prevalence of kinky tails increased to 12% and even to 28% in group 1 (relative tail length < 25.8%). This means that the piglets with the shortest tails were 5.6 times more likely to be accompanied by kinked tails than the piglets with the longest tails. This was more than the 4% described by [18]. The comparison of litters without kinked tails with litters in which at least individual kinks occurred showed no differences in tail lengths. At the same time, the tails in the litters with isolated kinks were significantly shorter in the piglets with kinks than in the piglets with normal tails. Thus, associations between tail length and kinks did not exist at the litter level, but only at the individual animal level. An even closer genetic association between the two traits could be inferred from this. The occurrence of kinks was further associated with certain boars and sows in both the present study and the study by [18]. The resulting genetic component was not the subject of the present study, but work in different animal species [19, 23, 25, 26, 31–34] shows associations between tail length and occurrence of kinks associated with different genes (e.g. [26]), often with the T gene, including pigs [19]. In some of these studies, further malformations in the spina and limb region were detected [24], which could not be systematically investigated in the present study. It can therefore be assumed that selection for shorter tails, in addition to the open question of efficiency with regard to biting and the lack of an approach to reducing the basic problem caused by husbandry, would also lead to an increased prevalence of malformations, at least in the tail, which would result in further damage in the affected animals. On the other hand, 74.3% of the 20% piglets with the shortest tails were not affected by kinked tails, so that with careful selection, shortened tails without such side effects might also be conceivable. Further research is required for this.

Recent studies have shown that inflammatory changes in the blood vessels at the base of the tail can also lead to clinical inflammation and necrosis at the tail base and tail tip by impairing the blood supply to the downstream tissues [28–30]. It was found that the clinical alterations at the base of the tail were histopathologically associated with vasculitis, intimal proliferation and thrombosis [29, 30], which restricted blood flow to the subsequent tissues of the tail [28]. As shortened tails and kinks are known to be associated with significant pathomorphological alterations in other species [24], it was hypothesised that the endogenous processes associated with shortened tails and kinks could exacerbate inflammation and/or necrosis of the tail by putting pressure on the blood vessels and thus, decreasing blood supply. In fact, signs of inflammation, especially in the form of bristle loss, redness and swelling at the tail base, already appeared in the 3-day-old suckling piglets. Only few piglets were affected by the more serious forms like exudation, necrosis and bleeding at the base of the tail, whereas the tip of the tail was affected in 2 to 37% of animals at both ages. The development of tail length from day 3 to day 39 could not be followed because the piglets had to be docked after the examination on day 3 due to high occurrence of tail biting/tail losses on the farm. The stumps were examined again on day 39, and inflammation and necrosis were also found in a similar distribution in the 39-day-old weaners after docking. While the inflammatory alterations occurred spontaneously in the suckling piglets, their expression in the weaners was artificially modified by docking, but in the same way in all piglets, regardless of tail length or kinks.

There was no association between tail length and inflammatory alterations. At the tail tip, exudation, necrosis and bleeding were also observed, especially in the stumps of the weaners, but again without significant influence of the tail length. Thus, the probability that genetic shortening of the tail per se might lead to increased susceptibility to inflammation and necrosis seems low. These findings essentially also apply to the issue of kinked tails, but in the 39-day-old animals there were indeed significantly more necroses associated with kinked tails than with intact tails at the tail base (2.4% vs. 0%, *P* = 0.02) and the stump (29.3% vs. 13.6%, *P* = 0.007). Comparable results are not available in the literature so far. The fact that the tails had to be docked is a weakness of the study in this case, because the docking was done proximal to the kink. Whether the changes at the tip of the tail would have been even more pronounced in kinked tails without docking remains open. The fact that despite docking, the kinked tails showed necrosis twice as often as normal tails could also be interpreted to mean that alterations could also be present proximal to the kink. However, further histopathological studies are needed to answer this question. It also remains to be clarified in further studies what the increased appearance of necrosis in kinked tails is based on and which pathohistological processes are responsible for this. The results suggest that kinked tails may be more prone to necrosis than normal tails, especially if the tails are exposed to further interventions or injuries (tail docking). However, the low correlation of tail length and kinky tails with the overall incidence of inflammation suggests only a slight association between these two aspects.

Inflammation and necrosis at tail base and tail tip are common, as expected, and their distribution is consistent with other studies [30, 35, 36], but their prevalence is not influenced by tail length or the occurrence of kinks, except for the necrosis of kink tails discussed above. Bleeding was found rather rarely. It could be associated with disturbances in blood homeostasis [37], rather than with short tails or kinks. Cases of tail biting were undetected in both suckling piglets and weaners, neither by the daily observation, nor by the evidence of biting marks.

## Conclusion

Pigs may show marked differences in relative tail length. It is not clear to what extent the decrease in biting attacks after shortening the tail is due to the presumed higher sensitivity of the stump or to the actual shortness of the tail. This leaves open whether selection for shorter tails could actually contribute to a significant decrease in tail biting and if the degree of shortness that would be needed could ever be reached. No studies on this are available so far. However, with increasing degrees of tail shortening, the probability for tail abnormalities in the form of kinked tails increases significantly. Besides higher frequencies of tail tip necrosis, an overall increased tendency for inflammation and necrosis was not found in suckling pigs and weaners in association with shorter tails nor with the occurrence of kinked tails. Based on the results obtained, any further efforts to shorten the tail should always take into account the possibility of an increased incidence of kinked tails and thus additional avoidable damage.

## Methods

### Clinical examination

A total of 348 piglets were available for the study. All piglets originated from routine matings for commercial piglet production at the “Oberer Hardthof” teaching and research station of the Department of Agricultural Sciences at the Justus Liebig University of Giessen. No targeted matings were made for the study. Piglets were merely measured, weighed, photographed, and subjected to zootechnical procedures: teeth clipping (day 1), ear tacked (day 1), iron application (Ursoferran, 200 mg/ml, Serumwerke Bernburg, Germany, day 2) and castration (day 6). All piglets were vaccinated against PCV2 and *Mycoplasma hyopneumoniae* (Porcilis PCV Mhyo, MSD, Munich, Germany) on day 25, three days before weaning. All experiments were approved by the responsible animal welfare officer of the Ethical Board of the Justus-Liebig-University Gießen, Germany with the reference JLU_kTV_4_2021. The work was compatible with the animal welfare guidelines of the Justus Liebig University and no ethical approval was necessary according to the German Animal Welfare Act.

All methods were carried out in accordance with relevant guidelines and regulations. The piglets came from 24 litters out of 6 Pietrain boars with 21 sows from the commercial pig producing program of the station. The sows were from four internal lines, crossed with Topigs Norsvin (TN70). The boars were mated to 1 up to 7 sows. Three sows had two litters with two different boars. Litter size was 17.54 ± 3.7 piglets in total and 16.7 ± 3.27 piglets born alive.

**Fig. 6:**
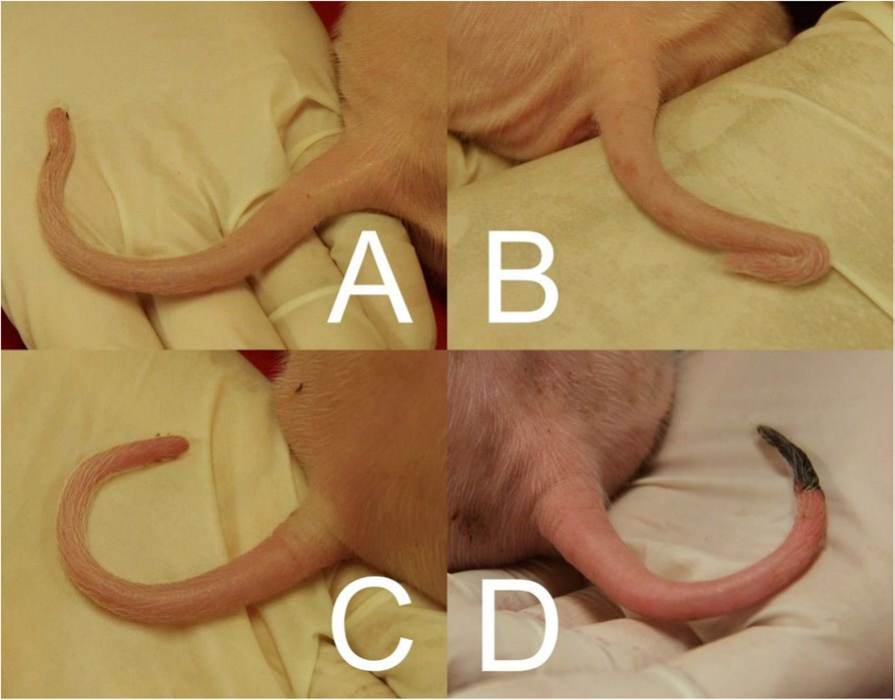
Clinical examination of the tails. Top row: slight kink (30°; **A** and strong kink (180°, **B** of the tail tip. Inflammation score for (**A**): tail base 1, tail tip 1; for (**B**): tail base 1, tail tip 1. Bottom row: bristle loss, swelling and redness (Score 3) (**C**) and tail tip necrosis (Score 4) (**D**)

All piglets were examined on day 3 of life. Tail length and body length were measured with a tape measure in cm with one decimal. Tail length was measured from the tip of the tail to its base on the body following the whole tail exactly in its median. Body length was measured from the upper ear base to the tail tip. Because the tail length depends primarily on the total length of the piglets and the piglet weights varied significantly, the relative tail length (tail length/body length × 100) was used as the measured value. Tails were photographed. Kinked tails were recorded (Fig. 6) and the degrees of the kinks were roughly categorised as 30°, 60°, 90°, and 180° from the photos. All observations were exclusively done by one experienced observer.

The presence of inflammation and necrosis at the base and tip of the tail (Fig. 6C, D), was also recorded by photography by the same observer. Signs of inflammation and necrosis were evaluated separately for base and tip characterized according to bristle loss (0/1), redness (0/1), swelling (0/1), exudation (0/1), necrosis (0/1), and haemorrhage (0/1). The findings were summed to give a score for tail base and tail tip, each ranging from 0 to 6.

Tail biting was not systematically assessed by continuous video analysis, but both suckling pigs and weaners were checked daily for signs of biting events like cross tissue damage or bleeding by the farmer and three times a week by the observer. In addition, all tails were systematically examined for bite marks (with counterbite) at days 3 and 39 by the same observer. All piglets were tail docked directly after examination on day 3, due to the high prevalence of tail lesions in the herd. For tail docking, the tail was measured and then exactly the last third was removed by cauterisation, independent of the presence and position of a kink. Therefore, the tail tip data for day 3 refers to the actual tip, the data for day 39 refers to the tail end created by tail docking. Otherwise, inflammation and necrosis were assessed on day 39 (11 days after weaning at 28 days) analogous to day 3, considering the same piglets.

### Statistics

Statistical analysis was done with the program package IBM-SPSS V27 (Munich, Germany). All data were analysed with descriptive statistics. The dependent variable was the relative tail length in %.

For the relative tail length, effects of boar (A-F), sow (*n* = 21) within line (1–4), sex (f,m), kinked tail (0, 1), together with weight at birth and number of piglets in the litter as covariables were considered applying a general linear model for any metric data.

The model was: Y_ijklm_ = µ + Boar_i_ + Sow(Line)_j_ + Sex_k_ + Kinked_l_ + b(Npiglet) + b(Wpiglet) + e_ijklm_

With

Y_ijklm_ = observed value

µ = mean value

Boar_i_ = fixed effect of the boar (I = A to F)

Sow(Line)_j_ = fixed effect of the sow (*n* = 21) within line (1–4)

Sex_k_ = fixed effect of the piglet’s sex (k = male, female)

Kinked_l_ = fixed effect of the tail (l = kinked versus not kinked)

b(Npiglets) = number of piglets in the litter as covariate

b(Wpiglet) = weight of piglet at birth as covariate

e_ijklm_ = residual variance

Binary data (kinked tail, inflammation data) and ordinal data showing the five degrees of kinks (0, 30, 60, 90, 180°) were analysed with chi square test and with monofactorial linear model using probit function, because the data distribution (few cases with kinked tails) led to empty fields and did not allow for reasonable multivariate analysis. The sows’ parity (1–4) was tested, but rejected from the model, because there was no effect. All data were Bonferroni-corrected. Regression and correlation coefficients were calculated with linear regression method.

Five groups were formed based on the tail length (tail length group), each including 20% of the piglets (percentiles). Group 1 contained the 20% of piglets with the shortest, group 5 those 20% with the longest tails. To analyse inflammation scores of tail base and tail tip, the above mentioned general linear model was applied, including tail length group (1–5). Correlations were calculated as Spearman correlations.

Associations of tail length or kinked tails with numbers of piglets, stillborn or living piglets and mummies were not predicted because of the small number of data arising from the 24 litters. The same applies to the aspect of survival rates from suckling piglets to weaners.

## Supplementary Information

Supplementary Material 1.

## Data Availability

The datasets used and analysed during the current study are available from the corresponding author on reasonable request.

## Abbreviation

SINS: Swine Inflammation and Necrosis Syndrome
