# Supplementary material for: Pig tail length is associated with the prevalence of tail malformations but not with inflammation of the tail
Source: BMC Vet Res. 2025 Feb 26;21:111. doi: 10.1186/s12917-025-04598-y (PMC11863879; doi:10.1186/s12917-025-04598-y)
Supplement: Supplementary file 1 — Supplementary Material 1. [file 12917_2025_4598_MOESM1_ESM.docx]

**Supplementary data to**

**Pig tail length is associated with the prevalence of tail malformations but not with inflammation of the tail**

Christiane Egerer^1^, Katharina Gerhards^1^, Sabrina Becker^1^, Petra Engel^2^, Sven König^2^ und Gerald Reiner^1*^

**Supplemental Table 1:** Signifikant differences between sows of figure 2 are given as black boxes.

|  | 743 | 745 | 782 | 793 | 810 | 838 | 858 | 902 | 906 | 921 | 951 | 957 | 958 | 959 | 962 | 963 | 964 | 968 | 992 | 999 | 1024 |
| --- | --- | --- | --- | --- | --- | --- | --- | --- | --- | --- | --- | --- | --- | --- | --- | --- | --- | --- | --- | --- | --- |
| 743 |  |  |  | 793 | 810 | 838 | 858 | 902 | 906 |  |  | 957 | 958 | 959 | 962 | 963 | 964 |  | 992 | 999 | 1024 |
| 745 |  |  | 782 |  | 810 | 838 |  | 902 |  |  |  | 957 | 958 | 959 | 962 | 963 | 964 |  | 992 |  | 1024 |
| 782 |  | 745 |  | 793 | 810 | 838 | 858 | 902 | 906 |  | 951 | 957 | 958 | 959 | 962 | 963 | 964 | 968 | 992 | 999 | 1024 |
| 793 |  |  | 782 |  | 810 |  |  | 902 |  | 921 | 951 | 957 | 958 | 959 | 962 |  | 964 | 968 | 992 |  | 1024 |
| 810 | 743 | 745 | 782 | 793 |  |  | 858 |  | 906 | 921 | 951 |  |  |  | 962 |  |  | 968 |  | 999 |  |
| 838 | 743 | 745 | 782 |  |  |  | 858 | 902 | 906 | 921 | 951 |  |  |  | 962 |  |  | 968 |  |  |  |
| 858 | 743 |  | 782 |  | 810 | 838 |  | 902 |  | 921 |  | 957 | 958 | 959 | 962 | 963 | 964 | 968 | 992 |  | 1024 |
| 902 | 743 | 745 | 782 | 793 |  | 838 | 858 |  | 906 | 921 | 951 |  |  |  |  | 963 |  | 968 |  | 999 |  |
| 906 | 743 |  | 782 | 793 | 810 | 838 |  | 902 |  |  |  | 957 | 958 | 959 | 962 | 963 | 964 |  | 992 |  | 1024 |
| 921 |  |  |  | 793 | 810 | 838 | 858 | 902 |  |  |  | 957 | 958 | 959 | 962 | 963 | 964 |  | 992 | 999 | 1024 |
| 951 |  |  | 782 | 793 | 810 | 838 |  | 902 |  |  |  | 957 | 958 | 959 | 962 | 963 | 964 |  | 992 | 999 | 1024 |
| 957 | 743 | 745 | 782 | 793 |  |  | 858 |  | 906 | 921 | 951 |  |  |  | 962 |  | 964 | 968 | 992 | 999 | 1024 |
| 958 | 743 | 745 | 782 | 793 |  |  | 858 |  | 906 | 921 | 951 |  |  |  |  |  |  | 968 |  | 999 |  |
| 959 | 743 | 745 | 782 | 793 |  |  | 858 |  | 906 | 921 | 951 |  |  |  | 962 |  |  | 968 |  | 999 |  |
| 962 | 743 | 745 | 782 | 793 | 810 | 838 | 858 |  | 906 | 921 | 951 | 957 |  | 959 |  | 963 | 964 | 968 | 992 | 999 | 1024 |
| 963 | 743 | 745 | 782 |  |  |  | 858 | 902 | 906 | 921 | 951 |  |  |  | 962 |  |  | 968 |  |  |  |
| 964 | 743 | 745 | 782 | 793 |  |  | 858 |  | 906 | 921 | 951 | 957 |  |  | 962 |  |  | 968 | 992 | 999 | 1024 |
| 968 |  |  |  | 793 | 810 | 838 | 858 | 902 |  |  |  | 957 | 958 | 959 | 962 | 963 | 964 |  | 992 | 999 | 1024 |
| 992 | 743 | 745 | 782 | 793 |  |  | 858 |  | 906 | 921 | 951 | 957 |  |  | 962 |  | 964 | 968 |  | 999 | 1024 |
| 999 | 743 |  | 782 |  | 810 |  |  | 902 |  | 921 | 951 | 957 | 958 | 959 | 962 |  | 964 | 968 | 992 |  | 1024 |
| 1024 | 743 | 745 | 782 | 793 |  |  | 858 |  | 906 | 921 | 951 | 957 |  |  | 962 |  | 964 | 968 | 992 | 999 |  |
